# Highlights of Current Dietary Guidelines in Five Continents

**DOI:** 10.3390/ijerph18062814

**Published:** 2021-03-10

**Authors:** Maria Luz Fernandez, Dele Raheem, Fernando Ramos, Conrado Carrascosa, Ariana Saraiva, António Raposo

**Affiliations:** 1 Department of Nutritional Sciences, University of Connecticut, Storrs, CT 06269, USA; 2 Northern Institute for Environmental and Minority Law (NIEM), Arctic Centre, University of Lapland, 96101 Rovaniemi, Finland; braheem@ulapland.fi; 3 Pharmacy Faculty, University of Coimbra, Azinhaga de Santa Comba, 3000-548 Coimbra, Portugal; framos@ff.uc.pt; 4 REQUIMTE/LAQV, Rua Dom Manuel II, Apartado 55142, 4051-401 Oporto, Portugal; 5 Department of Animal Pathology and Production, Bromatology and Food Technology, Faculty of Veterinary, Universidad de Las Palmas de Gran Canaria, Trasmontaña s/n, 35413 Arucas, Spain; conrado.carrascosa@ulpgc.es (C.C.); ariana_23@outlook.pt (A.S.); 6 CBIOS (Research Center for Biosciences and Health Technologies), Universidade Lusófona de Humanidades e Tecnologias, Campo Grande 376, 1749-024 Lisboa, Portugal

**Keywords:** dietary guidelines, Mediterranean, the USA, Japan, Argentina, South Africa

## Abstract

The dietary guidelines as well as the organizations that establish the recommendations are not homogeneous across regions of the world. Each country utilizes specific icons to better describe to the public easy ways to follow specific recommendations, including the use of pyramids, plates, and other forms of presenting key information. All dietary guidelines are updated within certain periods to ensure that new findings or specific changes are communicated to the public. The purpose of this commentary is to describe the most updated information as well as some history on how these symbols are utilized in different countries or areas of the world. The updated Mediterranean pyramid as well as MyPlate and the Pyramids utilized in South Africa, Japan, and Argentina are discussed in this commentary.

## 1. Introduction

The importance of communicating the dietary guidelines to the public in a comprehensive yet easy to follow approach is well-recognized [1]. Therefore, the need to identify the means to exemplify these guidelines led to the creation of dietary pyramids and MyPlate to better illustrate these recommendations. The first dietary pyramid was published in Sweden in 1974 followed by the United States (US) pyramid in 1992 [2]. The idea was to illustrate that foods that should be consumed in greater amounts should be located in the base of the pyramid, while those that should be eaten sparingly should be at the top [2]. Later, a number of countries decided to convey a similar message regarding specific guidelines, which followed cultural and regional food recommendations. An example is the Mediterranean Diet Pyramid, created in 1993, which illustrates the healthy food choices followed by the Mediterranean region.

Other dietary pyramids that were created early on and that have been recently updated are those from Argentina created in 2000, South Africa created in 2003, and Japan created in 2014. Recently, in June of 2011, The US Department of Agriculture (USDA) created MyPlate to have a better visualization of the recommended portions of different food groups including grains, vegetables, fruits, dairy, and high-protein foods [2]. 

The Food and Agriculture Organization (FAO) emphasized food-based dietary guidelines with key messages for many countries including USA, South Africa, Argentina, and Japan [3]. In this commentary, we have chosen to briefly discuss different representations of the updated dietary guidelines in five countries/regions. We highlight the way these recommendations are conveyed and briefly discuss their strengths and weaknesses.

## 2. Dietary Pyramid and My Plate

**Figure 1:**
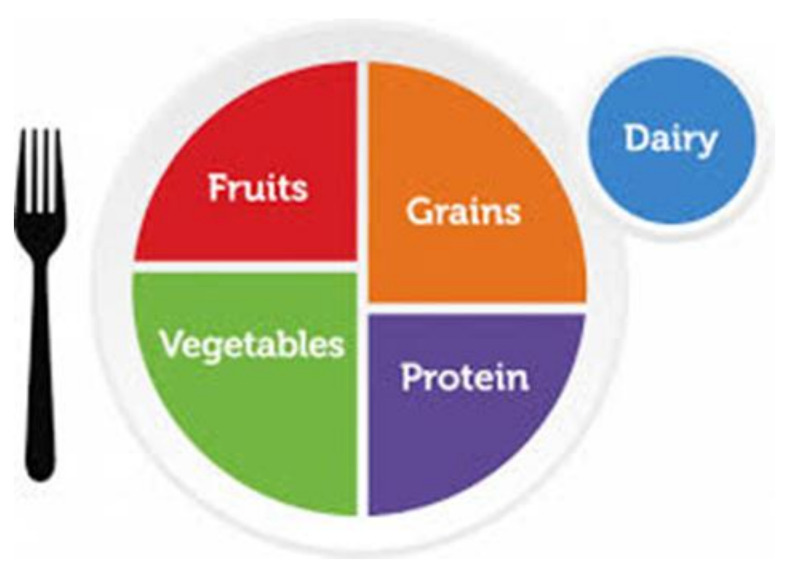
MyPlate [4].

MyPlate (Figure 1) was created to substitute the US Dietary Pyramid in 2011 resulting in the ending of 19 Dietary Pyramids since 1992 [4]. MyPlate is the representation of a plate setting with a glass that includes five groups: grains, vegetables, fruits, protein foods, and dairy. Several strengths have been identified in how MyPlate is represented: (1) MyPlate has been adapted to cover the needs of pregnancy and breastfeeding, toddlers, children, adults, and older adults and thus is more precise in its recommendations across the life cycle [4]; (2) the web site created by USDA also provides a list of recipes and innovative ways to follow MyPlate; (3) MyPlate also recommends moderate to vigorous physical activity 150 min per week [5]. Further, studies have shown that following MyPlate contributes to the maintenance of a healthy body weight plus a lower risk of hypertension in adults over 50 years of age [6]. MyPlate also recommends more satiating foods to treat obesity rather than caloric counting. The key messages from MyPlate are to follow a healthy eating pattern that supports adequate nutrient intake to prevent the risk of chronic disease; to focus on a variety of nutrient dense foods; to limit calories from added sugars, saturated fat, and sodium; and to shift to healthier beverage choices.

**Figure 2:**
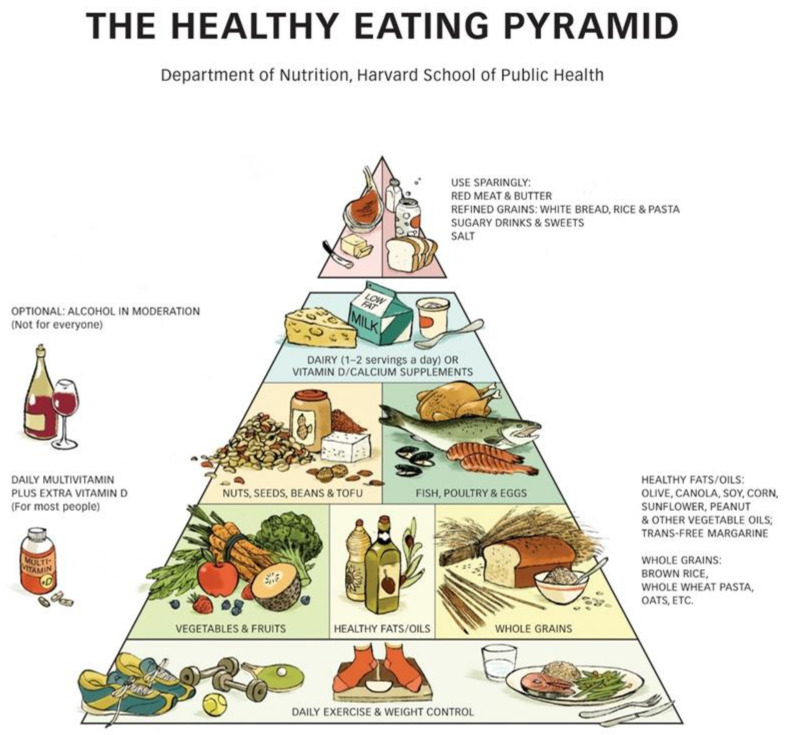
Harvard Dietary Pyramid [7]. Copyright © 2008. For more information about The Healthy Eating Pyramid, please see The Nutrition Source, Department of Nutrition, Harvard T.H. Chan School of Public Health, www.thenutritionsource.org (accessed on 1 February 2021), and Eat, Drink, and Be Healthy, by Walter C. Willett, M.D. and Patrick J. Skerrett (2005), Free Press/Simon & Schuster Inc.

Although MyPlate is the newest way by which USDA depicts dietary recommendations, the Pyramid is still used. There is a Harvard Dietary Pyramid (Figure 2), which in addition to including the type of foods that should be consumed most at the base of the pyramid also provides additional information regarding exercise and lifestyle practices [7]. This pyramid has several strengths that are worth mentioning: (1) the bottom of the pyramid, instead of including food groups, states the importance of both exercise and maintenance of a healthy body weight. (2) The food groups are arranged differently than in the original pyramid, since whole grains, healthy oils, fruits, and vegetables are recognized as the foods that should be consumed the most, followed by fish, poultry and eggs, nuts, seeds, and beans, of which equal intake is recommended; the next portion of the pyramid recommends dairy as well as Vitamin D and calcium supplements, and the tip of the pyramid includes red meat, butter, refined grains, sugar, and salt to consume in moderation. (3) Other recommendations include optional alcohol intake in moderation (not meant for all populations) and intake of daily multivitamin plus Vitamin D for most people.

These two approaches for dietary recommendations in US have a number of strengths in that they do not only focus on foods but also on maintenance of healthy body weight and exercise. However, they do not address sustainability or environmental concerns associated with food production.

## 3. Mediterranean Diet Pyramid

**Figure 3:**
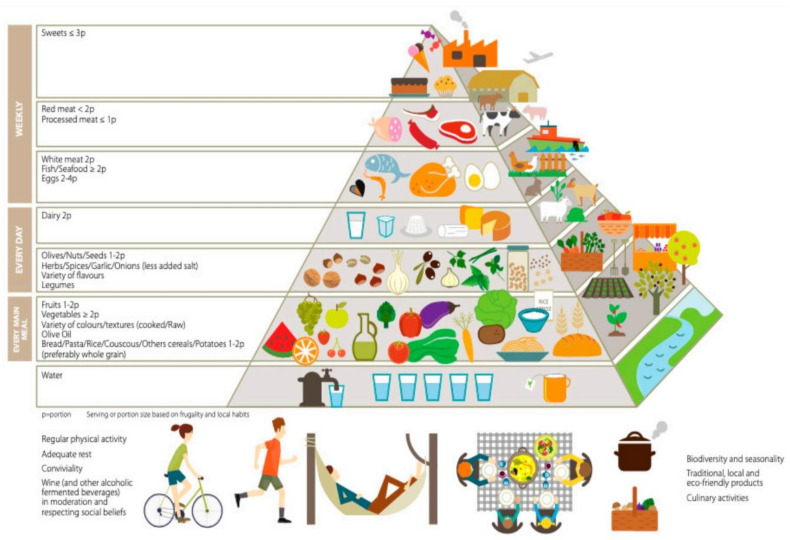
Updated Mediterranean Diet Pyramid [8].

Serra-Majem et al. recently reported the updated Mediterranean Diet Pyramid (MDP) (Figure 3) [8], a sequel of the previous study by Bach-Faig et al. [9], which summarized and updated the traditional Mediterranean Diet (MD) of those basin areas that have developed with modernization. This region includes all the countries that surround the Mediterranean Sea: France, Portugal, Italy Spain, Greece, Malta, and Cyprus, which is characterized by specific climate and by a biodiversity of species [10].

The MDP was a shared and dynamic cultural heritage recognized in 2010 by UNESCO [8]. This updated MDP highlights the fact that it takes into consideration not only nutrition but also sustainability and the environment as well as economic implications and sociocultural factors and comprises the combined effort of a number of expert individuals who updated the pyramid based on current challenges that Mediterranean populations are confronting [8]. This updated MDP constitutes a logical follow-up to the original MDP that was created in 1993 and updated in 2010 by a group of professionals and organizations who have dedicated many years to the evaluation and promotion of the MD [11]. Such collaborative efforts on the Med Diet 4.0 framework in the years 2015 and 2016 and the early part of 2020 led to a continuous update of the MDP. 

It is well established that the MD has been recognized as a healthy diet that not only protects against chronic disease including heart disease, Type-2 diabetes, and cancer, but also provides numerous healthy dietary components that promote health and well- being [12,13]. The updated pyramid provides very detailed information on the main food items that constitute the MD as well as a clear description of the different nutrients and antioxidants present in foods and their precise role in promoting health. A clear description of the portions that should be consumed either daily or weekly is also included, which are easy to follow and comprehend. In addition, authors rationalize that different countries adapt the components of the MD according to their own traditions, an example being the consumption of wine that is replaced by herbal teas in Muslim societies. Since a healthy dietary pattern needs to be linked to food production and sustainability [14,15], the updated MDP, in addition to the well-described nutritional components of the diet, also emphasizes exercise, conviviality, biodiversity, and culinary activities associated with the consumption of eco-friendly local seasonal products. 

**Figure 4:**
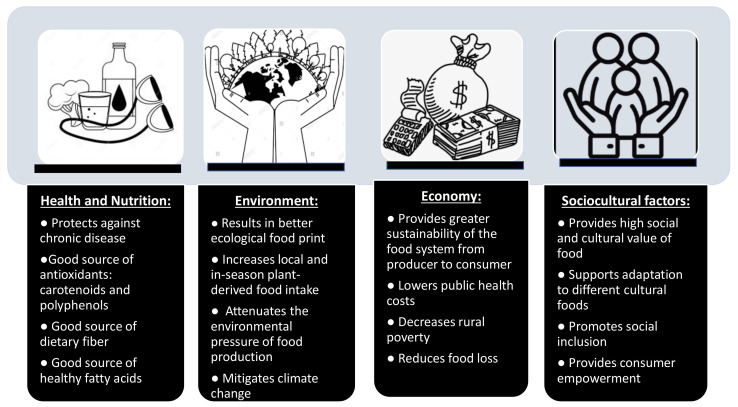
The updated Mediterranean Diet Pyramid is characterized for its contribution to Health and Nutrition, the Environment, the Economy and the incorporation of Sociocultural Factors. Some of the elements supporting these claims are depicted in this figure.

A summary of the salient points of this MDP are presented in Figure 4, which exemplifies the benefits of the Mediterranean diet for (1) nutrition and health, (2) the environment, (3) the economy, and (4) sociocultural factors.

The Mediterranean Diet (MD) is unique as it complies with at least 11 out of 17 goals of the United Nations Sustainable Development Goals (SDGs), mainly SDG2–SDG8 and SDG11–SDG15 [8], which are related to Zero Hunger, Good Health, Quality Education, Gender Equity, Clean Water, Affordable and Clean Energy, Economic Growth, Sustainable Cities and Communities, Responsible Consumption and Production, Climate Action, and Life Below Water [8]. 

## 4. South African Dietary Pyramid 

**Figure 5:**
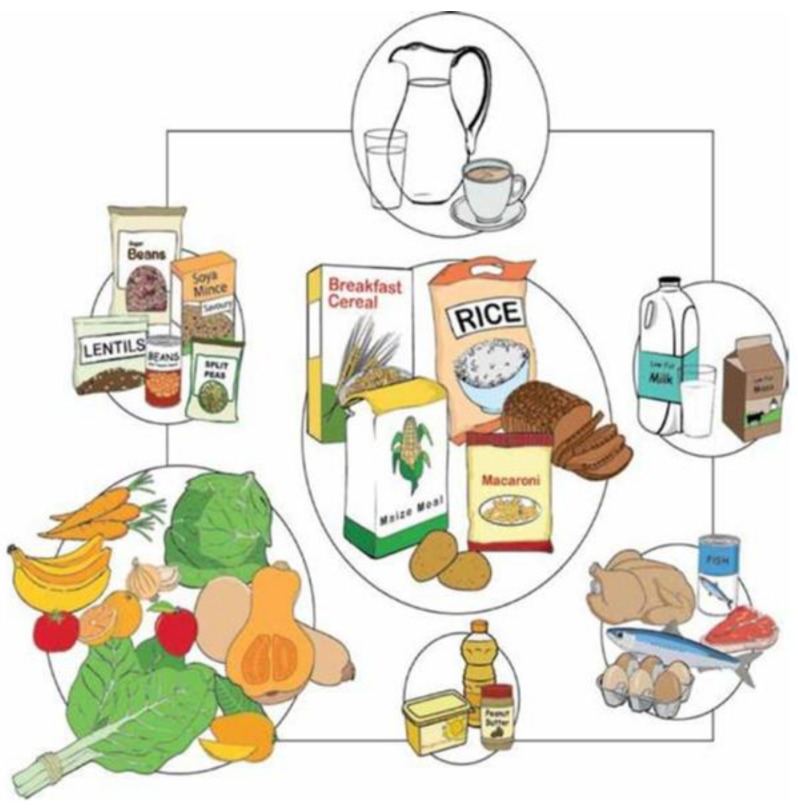
Food-based dietary guidelines (South Africa) [3].

South Africa first published food-based dietary guidelines (FBDG) in 2003. A revised version was launched in 2012 (Figure 5). Seven differently sized circles are used by the Circles of South Africa to “symbolically reflect the proportional volume that the group should contribute to the total daily diet” [16]. Each circle reflects a food group and healthy eating recommendations that are associated. In order to preserve good health, the South African model reflects what should be consumed, as opposed to what should be avoided. The key messages are related to specific foods consumed in that region, including starchy foods, dry beans, split peas, lentils, and soy, and it also emphasizes the consumption of fruit and vegetables. The South African Pyramid also recommends to drink milk every day as well as to consume fish, lean meat, and eggs daily while emphasizing that sugars, saturated fat, and salt should be eaten sparingly.

Food groups are not written on the FBDG image itself; however, they are otherwise indicated in the accompanying FBDG [16]. As exemplified by ethnically varied staples such as corn meal, rice, potatoes, and bread, the main group includes distinct grains and starches. These starchy foods make up the largest of the seven circles, a relatively large part of the proposed South African diet. In the next larger circle, fresh fruits and vegetables are pictured; the overall amount ingested should be somewhat equal to that of starches, since the circle is somewhat narrower. By analogy, legumes, animal proteins, and dairy products, as indicated by their smaller circle sizes, are to be eaten in comparatively smaller volumes. The South African model illustrates socio-economic facts and accessibility problems, as numerous items are sold in boxes, bags, cans, plastic jugs, and cartons, since not everybody can afford fresh ingredients. Fats and oils make up the smallest group, while water and tea are placed at the top. Compared to other dietary guidelines, the FBDG shows the relevance of daily water consumption by placing the image at the top of the graph. 

## 5. Argentinian Dietary Pyramid

**Figure 6:**
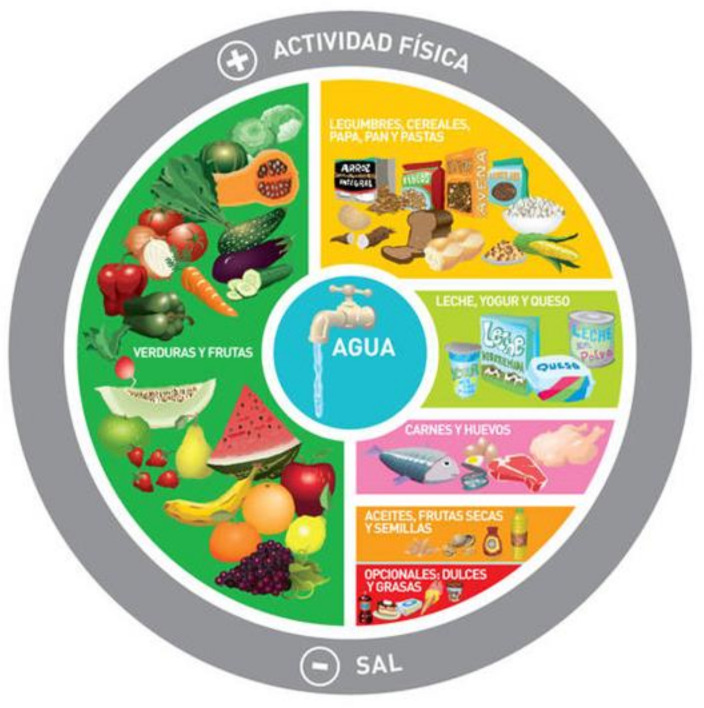
Food-based dietary guidelines—Argentina [3].

Argentina first launched its food-based dietary guidelines and food guide in 2000. They were revised in 2015 (Figure 6). The Dietary Guidelines for the Argentinian Population (DGAP) constitute a fundamental tool to promote the dissemination of knowledge. The DGAP are an educational instrument whose main aim is to promote more equitable intake of healthy food as well as improved dietary behaviors in the whole country. They translate the nutritional goals established for Argentina into practical messages, written in simple and understandable language. This pyramid, in addition to the recommendations of consuming five portions of fruits and vegetables, milk, yogurt, and cheese daily, has specific recommendations, including drinking eight glasses of water per day and 30 minutes of physical activity daily, plus it emphasizes that alcohol should not be consumed while driving and pregnant women and children should not drink alcohol.

The DGAP are also a planning tool for the sectors of health, education, production, industry, commerce, and all those who work in the food area [17]. The highlighted recommendations include the consumption of safe water, the practice of physical activity, the lower consumption of salt and a higher consumption of fibers, polyunsaturated fatty acids with a decrease in the consumption of saturated fats and sugars [17,18]. However, the Argentinian pyramid does not address sustainability or the environment.

## 6. Japanese Dietary Pyramid

**Figure 7:**
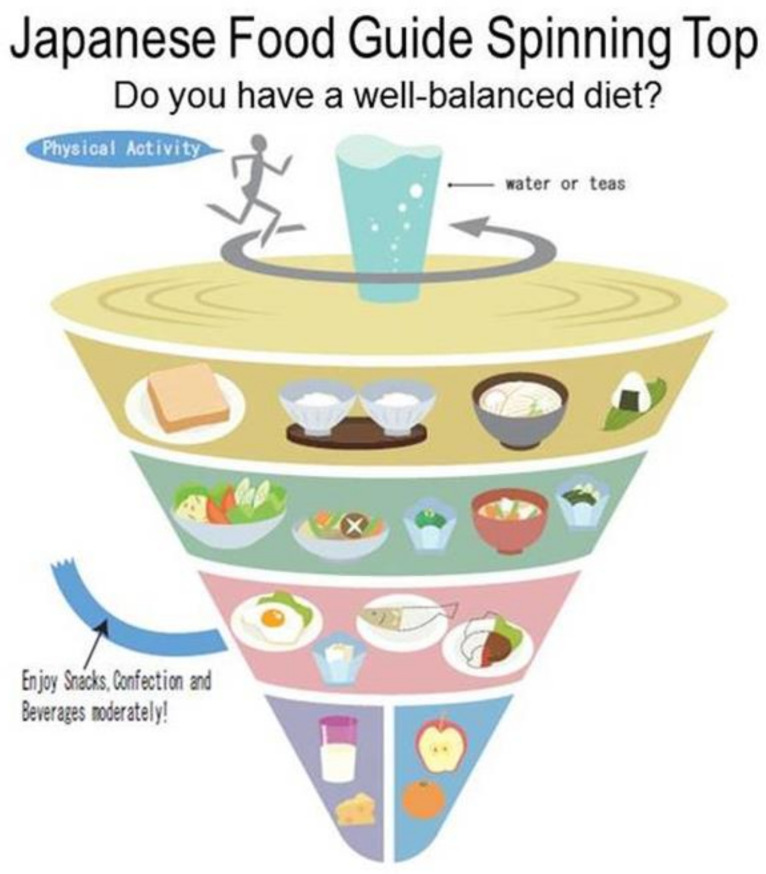
Japanese Food Guide Spinning Top [3].

In 2005, the Japanese government launched the Japanese Food Guide Spinning Top (JFG) to help Japanese citizens adopt nutritional recommendations to encourage healthier eating [19]. The JFG was revised in 2010 (Figure 7), and it is distinctive in that the quantity of food eaten in a daily diet is represented in amounts of the “dish” rather than in the “food” format and it uses the analogy of the shape of a spinning top, which reflects an element of Japanese culture [19].

The following categories of food are included in the JFG: grain dishes (rice, bread, noodles, etc.), vegetable dishes (vegetables, mushrooms, potatoes, and seaweed), fish and meat dishes (meat, fish, eggs, soybeans, etc.), milk (milk and milk products), fruit, confectionaries, sugar-sweetened drinks, and alcoholic drinks. The order of the food groups is determined by the size of the specified daily servings. It is advised to eat a combination of the food groups daily. There are special messages emphasized by this pyramid, including keeping regular hours for meals, enjoying the meals, maintaining a healthy body weight by physical activity, using proper cooking and storage methods, and tracking your food to monitor your diet.

The JFG is a valuable instrument for the assessment of Japanese people’s dietary consistency [20]. Previous investigations assessing the degree of compliance to the JFG (Food Guide score) have identified correlations between the Food Guide score and the risk of total mortality [21], depressive symptoms [22], and metabolic risk factors [23]. However, the JFG does not address environmental issues or take into consideration the economy.

## 7. Conclusions

In summary, all the updated pyramids and MyPlate provide more detailed information on nutrient consumption and food groups and include exercise as an important component, [3,4,8,16,17,19]. Further, they all cater to the specific socio-cultural factors of each country or region. However, most of the updated pyramids with the exception of the MDP do not address the environment or the sustainability of local production. The South African Pyramid does address the economy. The MDP addresses all these important points, resulting in a stronger message to consumers. It would be important that the US, Japan, Argentina, and South Africa update their recommendations, taking into consideration the environment and the economy. The major points addressed by of each of these pyramids is depicted in Table 1.

## Figures and Tables

**Table 1:** Points addressed by the Updated Pyramids from MyPlate and the Harvard Pyramid in the US, the Updated Mediterranean Pyramid (UMP), and the Argentinian and Japanese Pyramids.

Dietary Pyramids	Clear Dietary Recommendations	Physical Activity	Socio-Cultural Factors	Environment	Economy
MyPlate	Yes	Yes	Yes	No	No
Harvard Pyramid	Yes	Yes	Yes	No	No
UMP	Yes	Yes	Yes	Yes	Yes
South African Pyramid	Yes	Yes	Yes	No	Yes
Argentinian Pyramid	Yes	Yes	Yes	No	No
Japanese Pyramid	Yes	Yes	Yes	No	No
